# A Bayesian Mixture Modelling of Stop Signal Reaction Time Distributions: The Second Contextual Solution for the Problem of Aftereffects of Inhibition on SSRT Estimations

**DOI:** 10.3390/brainsci11081102

**Published:** 2021-08-21

**Authors:** Mohsen Soltanifar, Michael Escobar, Annie Dupuis, Russell Schachar

**Affiliations:** 1 Biostatistics Division, Dalla Lana School of Public Health, University of Toronto, 620, 155 College Street, Toronto, ON M5T 3M7, Canada; m.escobarr@utoronto.ca (M.E.); annie.dupuiss@mdstats.ca (A.D.); 2 The Hospital for Sick Children, Psychiatry Research, 4274, 4th Floor, Black Wing, 555 University Avenue, Toronto, ON M5G 1X8, Canada; russell.schacharr@sickkids.ca; 3 Department of Psychiatry, University of Toronto, 8th Floor, 250 College Street, Toronto, ON M5T 1R8, Canada

**Keywords:** reactive inhibition, stop signal reaction times, aftereffects of inhibition, mixture distribution, bayesian parametric approach

## Abstract

The distribution of single Stop Signal Reaction Times (SSRT) in the stop signal task (SST) has been modelled with two general methods: a nonparametric method by Hans Colonius (1990) and a Bayesian parametric method by Dora Matzke, Gordon Logan and colleagues (2013). These methods assume an equal impact of the preceding trial type (go/stop) in the SST trials on the SSRT distributional estimation without addressing the relaxed assumption. This study presents the required model by considering a two-state mixture model for the SSRT distribution. It then compares the Bayesian parametric single SSRT and mixture SSRT distributions in the usual stochastic order at the individual and the population level under ex-Gaussian (ExG) distributional format. It shows that compared to a single SSRT distribution, the mixture SSRT distribution is more varied, more positively skewed, more leptokurtic and larger in stochastic order. The size of the results’ disparities also depends on the choice of weights in the mixture SSRT distribution. This study confirms that mixture SSRT indices as a constant or distribution are significantly larger than their single SSRT counterparts in the related order. This result offers a vital improvement in the SSRT estimations.

## 1. Introduction

### 1.1. Reactive Inhibition

Inhibition refers to the ability to suppress actively, interrupt or delay an action [1]. Inhibition itself is a crucial dimension of executive control, which on its own is required for an organism to adjust behaviour according to changing conditions; this could be assessing inappropriateness of the current course of thought and action, changing goals or changing world [2,3,4]. Response inhibition is the ability to stop responses that are no longer appropriate [3]. Examples of response inhibition in daily life include braking quickly when driving into an intersection while another vehicle is running through a red light [4]. Two paradigms have been suggested to study response inhibition empirically in a laboratory setting: The Go/No-go task and the stop-signal task (SST). The later is widely used [1,5]. The stop-signal paradigm includes two response tasks: the go task and the stop task (e.g., stop 25% of the time). In go trials, the go reaction time (GORT) is the response to the stimulus such as “X” and “O” presented on the computer screen. In stop trials, the stop signal reaction time (SSRT) is the unobserved latency of the stopping response in the brain upon observing the stop signal (e.g., an auditory tone such as “beep”). The stop signal is presented to the participant after the passage of some time called the stop signal delay [6,7]. Often, the adjustment of stop signal delays (SSD or $T_{d}$) is made by the more reliable tracking method in which, depending on the previous trial’s success or failure, the $T_{d}$ is increased or decreased by 50 ms to achieve 50% overall successful inhibition at the end of the paradigm. In the go trials and stop signal response trials, the observed reaction times and the unobserved latency of the stopping response (i.e., GORT, SRRT and SSRT, respectively) are measured in milliseconds. In young adults trying to stop continuous actions, such as typing, the SSRT is close to 200 ms [8].

**Figure 1:**
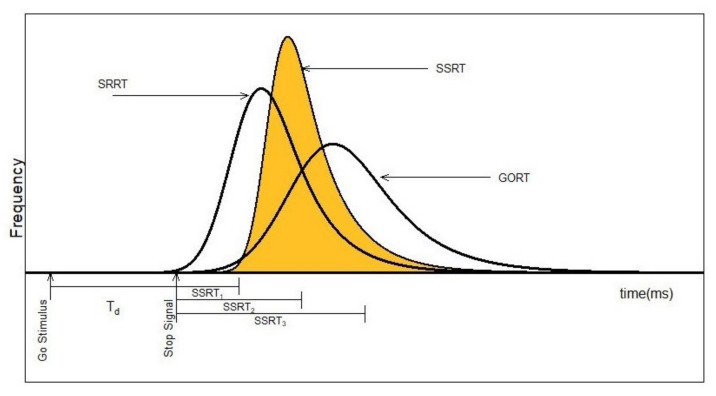
Graphical representation of the complete horse race model: GORT: go reaction times, SRRT: Signal respond reaction times, SSRT: Stop Signal reaction times, $T_{d}$: stop signal delay (SSD).

Several models have been proposed to evaluate and describe response inhibition in the stop-signal paradigm including the deadline model, the independent horse race model, the interactive horse race model and the Hanes–Carpenter model [9,10,11,12]. In this study, the independent horse race model is considered. It provides a theoretical framework in which researchers can measure the Stop Signal Reaction Times (SSRT) and its associated factors [13]. There are two types of the horse race model: the independent model with constant SSRT index and the complete independent model with non-constant SSRT distribution. In this study, we focus on the second model (see Figure 1). As the quantification index of the reactive inhibition, SSRT measurement has been a critical tool used by psychopathologists to make inferences about a patient’s ability to inhibit thought and action (i) on the spectrum of clinical groups (e.g., ADHD, OCD, autism and schizophrenia) and (ii) across various tasks and experimental conditions [13]. SSRT measurement’s precise estimation affects such inferences profoundly.

### 1.2. Estimation Methods: Context and Components

**Figure 2:**
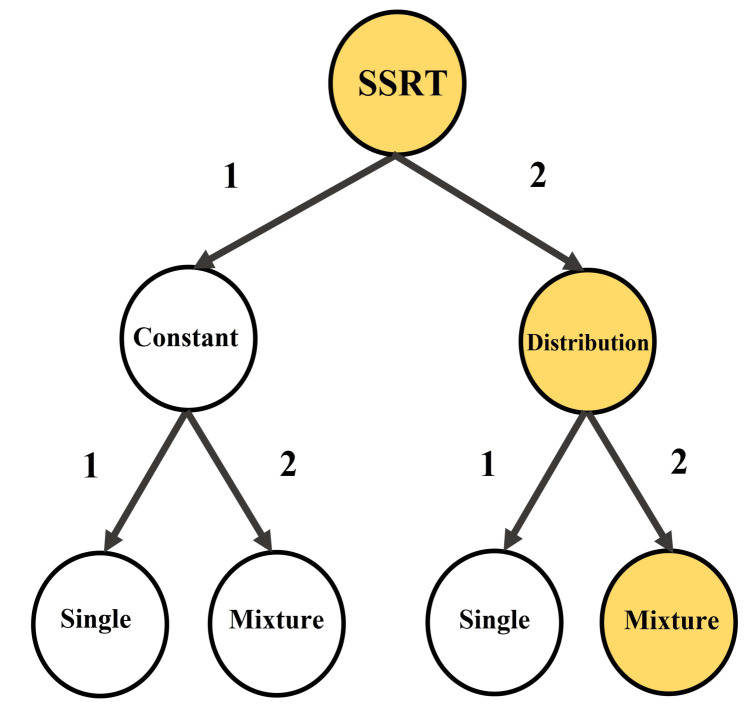
SSRT estimation in terms of context (constant/distribution) and components (single/mixture): current literature (path 1-1,1-2,2-1); this study (path 2-2).

There are several estimation methods of SSRT depending on two contexts in the SST literature: (i) as a constant index, or (ii) as a distribution of non-constant random variable. Within each context, these methods mentioned below refined the earlier proposed methods given their associated contexts (see Figure 2).

Referring to Figure 2 (path 1-1,1-2), there are four estimation methods of SSRT as a constant index: the mean crude method, the Logan 1994 integration method [3], the time series based state-space method [14] (path 1-1), and the weighted method and its mixture equivalent [15] (path 1-2) . Given a subject with go reaction time (GORT) random variable in the go trials with quantile function $Q_{GORT}$, *n* stop signal delays $T_{d},$ and the probability of successful inhibition(SI) denoted by $P(SI|\bar{T_{d}})$. Then, the first couple of the point indices of constant SSRT in the entire SST cluster are defined as in Equations (1) and (2) (The bar sign denotes average values.). Next, given higher reliability and less bias in the second index versus the first index, the second index has been recommended as the plausible index of constant SSRT [16]. Furthermore, one may transform raw trivariate SST time series data to trivariate state-space time series data using the missing data EM algorithm. Given the EM algorithm log-normally distributed outputs $GORT^{ss.ln}$ and $T_{d}^{ss.ln},$ the third point index of constant SSRT is defined as in Equation (3). The fourth index is essentially improvement of the second index under its associated contexts. For the last point index of constant SSRT, partitioning the entire SST cluster into two clusters of type-A SST cluster (trials following a go trial) and type-B SST cluster (trials following a stop trial) and calculating trial-type related Logan 1994 SSRT as $SSRT_{A}$ and $SSRT_{B}$ with corresponding weights $W_{A}=\#\mathrm{Type}\;A\;\mathrm{Stop}/\#\mathrm{Total}\;\mathrm{Stop},$ $W_{B}=1-W_{A},$ the last point index of constant SSRT is defined as in Equation (4):

(1) $$\begin{array}{ccc} SSRT_{Crude}^{c} & = & \bar{GORT}-\bar{T_{d}}, \end{array}$$

(2) $$\begin{array}{ccc} SSRT_{Logan1994}^{c} & = & Q_{GORT}\left(1-P\left(SI|\bar{T_{d}}\right)\right)-\bar{T_{d}}, \end{array}$$

(3) $$\begin{array}{ccc} SSRT_{SS.Logan1994}^{c} & = & Q_{GORT^{ss.ln}}\left(1-P\left(SI|\bar{T_{d}^{ss.ln}}\right)\right)-\bar{T_{d}^{ss.ln}}, \end{array}$$

(4) $$\begin{array}{ccc} SSRT_{Weighted}^{c} & = & W_{A}.SSRT_{A}+W_{B}.SSRT_{B}. \end{array}$$

Several researchers have shown that merely focusing on measures of central tendency in reaction times RT, including SSRT, gives insufficient information regarding the data’s nature. For instance, different clinical groups may have the same mean reaction times. However, the shape of their distributions differs in several aspects. The differences are in their tails, as seen in an ADHD group compared to the controls [17], or their domain of variance in a schizophrenia group versus controls [18]. These observations lead the researchers to study the entire SSRT distribution (Figure 2 (path 2-1,2-2)).

Referring to Figure 2 (path 2-1), there are two main methods to estimate SSRT as a single random variable: Colonius’s nonparametric method [19] and the Bayesian parametric method [20,21]. The first method retrieves the cumulative distribution function of SSRT given several components as follows: (i) go reaction times GORT in go trials with density $f_{GORT}$, (ii) signal respond reaction times SRRT in the failed stop trials with density $f_{SRRT}$, (iii) n stop signal delays $T_{d},$ and (iv) probability of successful inhibition (SI). The cumulative distribution function is calculated as in Equation (5) [19]. Although the first method theoretically gives the entire nonparametric distribution of SSRT, it cannot be implemented for empirical data in practice. It requires an unrealistically high number of trials for accurate estimations [20,21]. In the case of such estimations for simulated data, it has underestimated the mean of SSRT and overestimated the variance of SSRT [4,22]. These observations led researchers to propose the second method of estimation of SSRT in the Bayesian context under given parametric distributional assumptions for the involved GORT, SRRT and SSRT in the SST data [20]. The Bayesian Parametric Approach (BPA) presents a novel parametric approach to estimate the entire distribution of SSRT, which applies to real data with a low trial number [21]. Depending on the individual or hierarchical data, the BPA estimates parameters of the SSRT distribution distinctively. The estimation is done separately for each participant (called individual BPA or IBPA) or is done successively for each participant, and then the entire population (called hierarchical BPA or HBPA) [23,24]. Several studies have used the BPA approach in estimating SSRT distribution parameters for the case of Ex-Gaussian (ExG) distribution assumption with θ=(μ,σ,τ). For example, it has been shown that more practice in stop trials corresponds to lower estimated μ and higher estimated τ, for the SSRT ExG distribution [25]. Next, the BPA approach has shown that tyrosine consumption corresponds with lower estimated μ for the SSRT distribution [26]. Finally, the mixture BPA approach has been used to show the existence of trigger failures (Trigger Failure (TF) refers to the situation in which the participant fails to correctly diagnose and interpret the stop signal leading to his inability to attempt to inhibit the ongoing go process [27]). on stop signal performance in healthy control participants in two studies of inhibition deficiency in schizophrenia [28]. Assuming that the distribution of GORT and SSRT follows a parametric form, such as Ex-Gaussian (ExG) with parameter θ=(μ,σ,τ), the BPA estimates the posterior distributions $\pi \left(\mu_{stop}|data\right),\pi \left(\sigma_{stop}|data\right),$ and $\pi (\tau_{stop}|data).$ Then, the cumulative distribution function is calculated as in Equations (6) and (7) [21]:

(5) $$\begin{array}{ccc} F_{SSRT}\left(t\right) & = & 1-\left(1-P\left(SI|T_{d}\right)\right)\times \left(\frac{f_{SRRT}\left(t+T_{d}|T_{d}\right)}{f_{GORT}\left(t+T_{d}\right)}\right),\;\;0<t,T_{d}<\infty , \end{array}$$

(6) $$\begin{array}{ccc} F_{SSRT}\left(t\right) & = & F_{ExG}\left(t|\theta_{stop}=\left(\mu_{stop},\sigma_{stop},\tau_{stop}\right)\right),\;\;0<t<\infty ,\\ & & \mu_{stop}=E\left(\pi \left(\mu_{stop}|data\right)\right),\\ & & \sigma_{stop}=E\left(\pi \left(\sigma_{stop}|data\right)\right), \end{array}$$

(7) $$\begin{array}{ccc} & & \tau_{stop}=E\left(\pi \left(\tau_{stop}|data\right)\right). \end{array}$$

### 1.3. SSRT Estimation and Aftereffects of Inhibition: Constant vs. Distribution

#### 1.3.1. The Assumption

However, as mentioned by Logan [4], there is little known about the inhibition’s aftereffects and the type of questions of interest. One related unanswered question is whether there exist any aftereffects of the non-inhibited (e.g., go) trials and inhibited (e.g., stop) trials on inhibition and, in case of affirmative answer, how to measure SSRT as a constant or as a random variable. Here, in both the nonparametric and Bayesian parametric methods mentioned above, there is an implicit assumption. The assumption is about the aftereffects of go trials and stop trials in SST data; that is, the impact of the preceding trial type, either go or stop, on the current stop trial SSRT is assumed to be the same. Most of the SST literature has taken this assumption for granted. To the best of the authors’ knowledge, few studies have dealt with this question and estimated the SSRT distribution when this assumption is relaxed. Some studies have shown that after a go trial, the participants have a lower go reaction time GORT versus after the stop trial [29]. This phenomenon implies that the GORT distribution after each type of trial (go/stop) will differ, impacting the participant’s ability to stop after each trial type [30].

#### 1.3.2. Constant Index

There are only two studies in the SST literature that partially answered this question when SSRT is considered as constant index [14,15]. Here, it was shown when considering SST data in a longitudinal context $SSRT_{Weighted}^{c}>SSRT_{Logan1994}^{c};$ and when considering SST data in a missing time series context $SSRT_{\left(SS.Logan1994\right)}^{c}>SSRT_{Logan1994}^{c}.$ Both studies’ results were valid for the empirical SST data and the simulated SST data.

#### 1.3.3. Motivation

Given that constant SSRT index can be considered as a degenerate random variable, the above results partially shed light on the proposed question for the case of a non-degenerate SSRT random variable. However, it is still unknown whether the constant index related results hold for general SSRT random variable, in which order context these comparisons over random variables can be conducted and in which mechanism the pairwise comparisons of the involved paired sets of random variables is conducted.

### 1.4. Study Outline

This study offers an estimation of the SSRT distribution given the relaxed assumption of equal impact of the preceding trial type (go/stop) on the current stop trial SSRT distribution. It uses the notion of two-state mixtures [31] and proposes parametric mixture Bayesian modelling on the entire SST data set (Figure 2 (path 2-2)). The study’s outline is as follows: First, as in [15] for each participant, the overall empirical SST data is partitioned into type-A cluster SST data and type-B cluster SST data. Using the IBPA method, the fitted SSRT ex-Gaussian parameters are calculated for the cluster type SSRT distributions and the single SSRT distribution. The study’s empirical data provide an example of the violated assumption. Second, a mixture SSRT random variable is introduced as a natural generalisation of two cases: (i) its constant index $SSRT_{Weighted}^{c}$ counterpart in Equation (3) and (ii) its Bayesian parametric form under the ex-Gaussian distributional assumption. Then, considering the mean of posterior parameters as their point estimations, the key descriptive and shape statistics of the mixture SSRT $\left(SSRT_{Mixture}\right)$ are compared with those of type-A SSRT $\left(SSRT_{A}\right)$, type-B SSRT $\left(SSRT_{B}\right)$ and the single SSRT $\left(SSRT_{Single}\right)$. Third, we compare the involved pairs of distributions in usual stochastic order $\left({<}_{st}\right)$ at the individual and population level. The population-level comparisons use our proposed Two-Stage Bayesian Parametric Approach (TSBPA) and our proposed Paired Samples Parametric Distribution Test (PSPDT). Finally, the earlier comparisons are repeated and discussed in terms of the involved weights in the definition of proposed mixture SSRT $(SSRT_{Mixture}).$

## 2. Materials and Methods

### 2.1. The Data and Study Design

This study’s data and design are previously described in [32]. The study included 16,099 participants aged 6 to 19 years old and was conducted at the Ontario Science Centre in Toronto, Canada, between June 2009 and September 2010. Each participant sat in front of a computer screen with a game pad device equipped with two buttons (X/O) in their hands. The trials were either go or stop. Every go trial began with a 500 ms fixation point followed by a stimulus: An O or X presented for 1000 ms in the centre of a computer screen. The participants were instructed to press the correct button as fast as possible and the computer program would record their reaction times. Every stop trial began as the go trial with one extra feature: With an initial stop signal delay $T_{d}$ of 250 ms after the go stimulus, each stop trial included an audio stop signal cue (i.e., saying “Stop !”) presented through headphones to the participant in the context of the tracking method. The participants were supposed not to press the button (X/O) on the game pad. In the case of failed inhibition, the computer program would record the participants’ signal respond reaction times (SRRT). The entire SST dataset of the trials (go/stop) was recorded in a longitudinal form (see Appendix A). In this study, each participant completed four blocks of 24 trials with a random 25% stop signal trials in each block. There were 96 trials in total (24 stop signals and 72 go trials).

### 2.2. The Sample and Variables

#### 2.2.1. Cluster Type SST Data

In this study, four types of SST data clusters were identified as shown in Table 1 [15]:

1 Type-A SST data cluster: all trial preceded by go trials (See Appendix A)
2 Type-B SST data cluster: all trials preceded by stop trials (See Appendix A)
3 Type-S Single SST data cluster: all trials when considered for one single SSRT distribution
4 Type-M Mixture SST data cluster: all trials when considered for the mixture SSRT distribution.

A random sample of 44 participants was selected for further analysis. The entire stop signal task data for each participant was partitioned to type-A and type-B cluster types (see Appendix A). These participants each had a minimum of 10 type-B stop trials. For each participant, the above four types of SST data clusters were considered. Using IBPA, the corresponding ex-Gaussian SSRTs’ parameters θ=(μ,σ,τ) for type-A, type-B and type-S clusters were calculated as the means of posterior distribution estimation of the parameter in Equation (7) (see Appendix B).

#### 2.2.2. Ex-Gaussian Random Variable

Heathcote (1996) [33] formulated the ex-Gaussian (ExG) distribution with parameters (μ,σ,τ) with density given by

(8) $$f_{ExG}\left(t|\mu ,\sigma ,\tau \right)=\frac{1}{\tau }exp\left(\frac{\mu -t}{\tau }+\frac{\sigma^{2}}{2\tau^{2}}\right)\times \Phi \left(\frac{\mu -t}{\sigma }-\frac{\sigma }{\tau }\right):\sigma ,\tau >0,-\infty <t<\infty$$

where Φ is the standard normal cumulative distribution function. The first four non-central moments are given by

(9) $$\begin{array}{ccc} E(ExG) & = & \mu +\tau ,\\ E(ExG^{2}) & = & \mu^{2}+2\mu \tau +\sigma^{2}+2\tau^{2},\\ E(ExG^{3}) & = & \mu^{3}+3\mu \sigma^{2}+6\mu \tau^{2}+3\mu^{2}\tau +3\sigma^{2}\tau +6\tau^{3},\\ E(ExG^{4}) & = & \mu^{4}+4\mu^{3}\tau +6\mu^{2}\sigma^{2}+12\mu^{2}\tau^{2}+24\mu \tau^{3}+12\mu \sigma^{2}\tau +3\sigma^{4}+12\sigma^{2}\tau^{2}+24\tau^{4}. \end{array}$$

Finally, this random variable is right-skewed and leptokurtic with the following variance, skewness and kurtosis shape statistics:

(10) $$\begin{array}{ccc} Var(ExG) & = & \sigma^{2}+\tau^{2}\\ \gamma_{ExG} & = & 2{\left(1+\sigma^{2}\tau^{-2}\right)}^{\frac{-3}{2}}\\ \kappa_{ExG} & = & 3\left(\frac{1+2\sigma^{-2}\tau^{2}+3\sigma^{-4}\tau^{4}}{{\left(1+\sigma^{-2}\tau^{2}\right)}^{2}}\right). \end{array}$$

#### 2.2.3. Mixture SSRT Random Variable

Given single SSRT by $SSRT_{S}$, type-A SSRT by $SSRT_{A}$, type-B SSRT by $SSRT_{B}$ and $W_{A}∼Bernoulli\left(W_{A}^{c}\right)$ where the type A trial type weight $W_{A}^{c}$ is given by $W_{A}^{c}=\#\mathrm{Type}\;A\;\mathrm{Stop}/$#TotalStop, $W_{B}^{c}=1-W_{A}^{c},$ the Single SSRT and Mixture SSRT random variables were defined as follows (Note that with notation SSRT for random variable SSRT and $SSRT^{c}$ for constant SSRT estimated with frequentist methods, we have $E\left(SSRT\right)=SSRT^{c}$. Consequently, definitions in Equation (11) are natural generalisations of constant SSRT estimations with frequentist methods [15] to general non-constant random variables. Here onward, $W_{A}$ given the context is either a Bernoulli random variable or a constant number $W_{A}^{c}$ defined as above.):

(11) $$\begin{array}{ccc} SSRT_{Single} & {=}^{d} & SSRT_{S},\\ SSRT_{Mixture} & {=}^{d} & W_{A}\times SSRT_{A}+W_{B}\times SSRT_{B}. \end{array}$$

**Figure 3:**
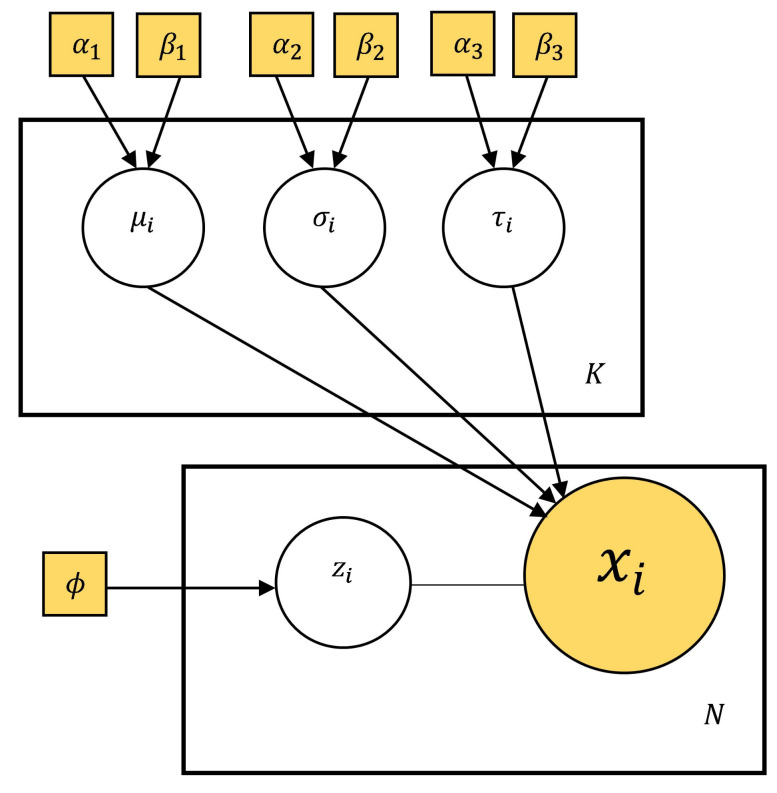
Bayesian Mixture Ex-Gaussian SSRT model using plate notation. Filled in shapes indicate known values.

In the Bayesian context and using IBPA and under Ex-Gaussian parametric assumption, we have $SSRT_{S}∼ExG\left(\theta_{S}=\left(\mu_{S},\sigma_{S},\tau_{S}\right)\right),$ $SSRT_{A}∼ExG\left(\theta_{A}=\left(\mu_{A},\sigma_{A},\tau_{A}\right)\right)$ and $SSRT_{B}∼ExG\left(\theta_{B}=\left(\mu_{B},\sigma_{B},\tau_{B}\right)\right),$ where the parameter point estimations are the means of the associated posterior distributions in IBPA. The Bayesian Mixture Ex-Gaussian SSRT model can be formulated as follows. The priors in the IBPA have uninformative uniform distribution and their own chosen parameters (α,β) are based on the positive ranges of parameters (μ,σ,τ) of the associated ExG distribution. Figure 3 presents the model using plate notation. Here, we have the following:

K=2: Number of cluster types,
N=96: Number of trials in SST data,
$\theta_{i}=\left(\mu_{i},\sigma_{i},\tau_{i}\right)$: Parameters of Ex-Gaussian SSRT distribution of the ith cluster (i=1:A;i=2:B),
$\mu_{i}∼U\left[\alpha_{1},\beta_{1}\right],\left(i=1:A;i=2:B\right):$ Here: $\alpha_{1}=10,\beta_{1}=2000,$
$\sigma_{i}∼U\left[\alpha_{2},\beta_{2}\right],\left(i=1:A;i=2:B\right):$ Here: $\alpha_{2}=10,\beta_{2}=2000,$
$\tau_{i}∼U\left[\alpha_{3},\beta_{3}\right],\left(i=1:A;i=2:B\right):$ Here: $\alpha_{3}=10,\beta_{3}=2000,$
$\phi =(\phi_{1},\phi_{2}):$ Prior Probability of clusters $(\phi_{1}=W_{A},\phi_{2}=W_{B}),$
$z_{i}∼Bernoulli\left(W\right):\;\;W=W_{A},$
$x_{i}$: ith SST trial,
$x_{i}{|}_{stop}∼ExG\left(\mu_{z_{i}},\sigma_{z_{i}},\tau_{z_{i}}\right).$

The first four moments of the Mixture SSRT are as follows:

(12) $$E\left(SSRT_{Mixture}^{k}\right)=W_{A}E\left(SSRT_{A}^{k}\right)+W_{B}E\left(SSRT_{B}^{k}\right):\;\;1\leq k\leq 4.$$

Consequently, the variance, the skewness and the kurtosis of the Mixture SSRT are computed by

(13) $$\begin{array}{cccc} \; & Var(SSRT_{Mixture}) & = & W_{A}E\left(SSRT_{A}^{2}\right)+\left(1-W_{A}\right)E\left(SSRT_{B}^{2}\right)\\ \; & \; & & -{\left(W_{A}E\left(SSRT_{A}\right)+\left(1-W_{A}\right)E\left(SSRT_{B}\right)\right)}^{2},\\ \; & \gamma_{SSRT_{Mixture}} & = & \frac{1}{Var^{\frac{3}{2}}\left(SSRT_{Mixture}\right)}(E\left(SSRT_{Mixture}^{3}\right)\\ \; & \; & & -3E\left(SSRT_{Mixture}\right)E\left(SSRT_{Mixture}^{2}\right)+2E^{3}\left(SSRT_{Mixture}\right)),\\ \; & \kappa_{SSRT_{Mixture}} & = & \frac{1}{Var^{2}\left(SSRT_{Mixture}\right)}(E\left(SSRT_{Mixture}^{4}\right)-4E\left(SSRT_{Mixture}\right)E\left(SSRT_{Mixture}^{3}\right)\\ \; & \; & & +6E^{2}\left(SSRT_{Mixture}\right)E\left(SSRT_{Mixture}^{2}\right)-3E^{4}\left(SSRT_{Mixture}\right)). \end{array}$$

**Remark** **1.** *Using new Equation (11) for SSRT, Colonious’s proposed nonparametric method for retrieving the entire SSRT CDF for given type-A weight $W_{A},$ type A delay $T_{dA}$, type-A signal respond density $f_{SRRTA}$, type-A GORT density $f_{GORTA}$, type-A probability of successful inhibition $P(SI|T_{dA})$ and the corresponding type B information yields the following mixture form:*

(14) $$\begin{array}{ccc} F_{SSRT}\left(t\right) & = & 1-W_{A}\left(\left(1-P\left(SI|T_{dA}\right)\right)\times \left(\frac{f_{SRRT}\left(t+T_{dA}|T_{dA}\right)}{f_{GORT}\left(t+T_{dA}\right)}\right)\right)\\ & & -W_{B}\left(\left(1-P\left(SI|T_{dB}\right)\right)\times \left(\frac{f_{SRRT}\left(t+T_{dB}|T_{dB}\right)}{f_{GORT}\left(t+T_{dB}\right)}\right)\right),\\ & & \;0<t,T_{dA},T_{dB}<\infty . \end{array}$$

**Remark** **2.** *The mixture modelling for SSRT proposed here can be applied with other non-Ex-Gaussian parametric RT distributions such as Ex-Wald, Wald, [34] Gamma, Weibull and Lognormal [35,36] with the required modifications in estimations.*

### 2.3. Statistical Analysis

For each participant IBPA under Ex-Gaussian parametric distribution was run three times: one for its associated cluster type-A, cluster type-B and single type-S SST data (a total of 132 times). We then calculated the mean posterior estimates of $\theta_{S}=\left(\mu_{S},\sigma_{S},\tau_{S}\right),$ $\theta_{A}=\left(\mu_{A},\sigma_{A},\tau_{A}\right),$ and $\theta_{B}=\left(\mu_{B},\sigma_{B},\tau_{B}\right).$ Then, the parameters, the descriptive statistics and the shape statistics for type-A SSRT $(SSRT_{A}),$ type-B SSRT $(SSRT_{B}),$ type-S single SSRT $\left(SSRT_{Single}\right)$ and type-M Mixture SSRT $\left(SSRT_{Mixture}\right)$ were calculated. The next steps of the analysis depended to the context and procedure described in the following.

#### 2.3.1. Comparisons Context: Real Numbers and Random Variables

Two sets of comparisons were conducted: (i) within a real numbers contexts and (ii) within a real-valued random variables context. For the first set of comparisons, paired *t*-test (PROC TTEST, ‘SAS/STAT’ software version 9.4 [37]) were conducted. These comparisons were made for the Ex-Gaussian distribution’s fitted parameters, the descriptive summary statistics and the shape statistics in the usual real numbers order (<) across cluster types. For the second set of comparisons, the two samples Kolmogorov–Smirnov (KS) tests (ks.test package stats, ‘R’ software version R.3.4.3 [38]) were conducted. These tests were conducted under the assumption of 96 points for the involved random variables CDFs to compare the SSRT random variables in usual stochastic order $\left({<}_{st}\right)$ across cluster types. Such test at the individual is

(15) $$\begin{array}{ccc} H_{0} & : & SSRT_{Single}\left(\vec{\theta_{S}}\right){=}_{st}SSRT_{Mixture}\left(\vec{\theta_{M}}\right),\\ H_{1} & : & SSRT_{Single}\left(\vec{\theta_{S}}\right)\neq_{st}SSRT_{Mixture}.\left(\vec{\theta_{M}}\right) \end{array}$$

#### 2.3.2. Comparisons Procedure: Random Variables

Given two sets of stop signal reaction times distributions ${\left\{SSRT_{Single}\left(\theta_{S_{k}}\right)\right\}}_{k=1}^{44}$ and ${\left\{SSRT_{Mixture}\left(\theta_{M_{k}}\right)\right\}}_{k=1}^{44}$, our problem of interest was an overall comparison between these two groups of distributions in usual stochastic order ${<}_{st}$ [39]. Our proposed problem was dealt with in two steps as follows: 

**Step** (1): Two-Stage Bayesian Parametric Approach (TSBPA) 

This proposed analysis is neither completely hierarchical Bayesian analysis nor completely conventional meta-analysis. It has components of both methods. On the one hand, it has two separates one-stage Bayesian analyses. On the other hand, it calculates overall population-level estimates in the second analysis with consideration of non-zero correlations. Referring to Equation (11), we define overall SSRT distributions per single S cluster type and mixture M cluster type as the following:

(16) $$\begin{array}{ccc} SSRT_{O.Single}\left(\vec{\theta_{S}}\right) & = & SSRT_{Single}\left(\bar{\theta_{S}}\right),\\ SSRT_{O.Mixture}\left(\vec{\theta_{M}}\right) & = & SSRT_{Mixture}\left(\bar{\theta_{M}}\right). \end{array}$$

where $\vec{\theta_{S}}=\bar{\theta_{S}}=\bar{\theta_{T}}=\left(\bar{\mu_{T}},\bar{\sigma_{T}},\bar{\tau_{T}}\right)$ and $\vec{\theta_{M}}=\bar{\theta_{M}}=\left(\bar{W_{A}},\bar{\theta_{A}},\bar{\theta_{B}}\right)$ with $\bar{W_{A}}=\sum_{k=1}^{44}W_{A_{k}}/44$ and $\bar{\theta_{A}}=\left(\bar{\mu_{A}},\bar{\sigma_{A}},\bar{\tau_{A}}\right),\bar{\theta_{B}}=\left(\bar{\mu_{B}},\bar{\sigma_{B}},\bar{\tau_{B}}\right)$ being computed by a Two-Stage Bayesian Parametric Approach (TSBPA) method described as follows.

**Figure 4:**
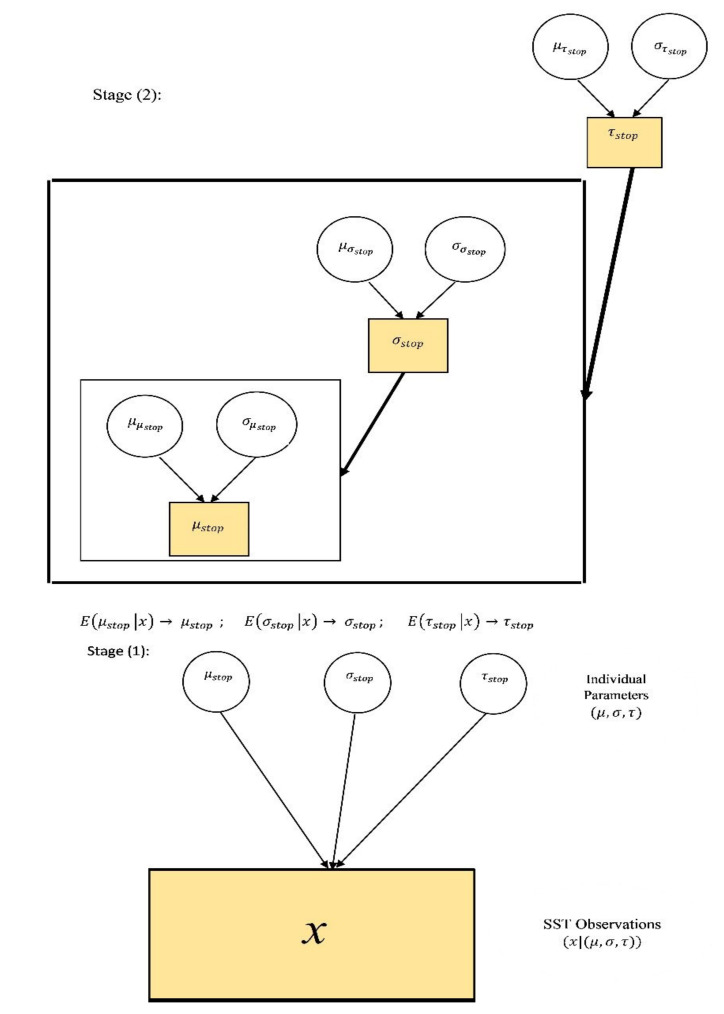
Two -Stage Bayesian Parametric Approach (TSBPA) with ex-Gaussian distributional assumption framework. Filled in shapes indicate known values.

In the TSBPA (See Figure 4), the data, the priors and the posterior estimations are considered as below [40,41,42]. We conduct the first stage with 3 chains, 5000 burn in out of 20,000 simulations in BEESTS 2.0 software. Then, we consider the mean of posterior estimates $\mu_{stop},\sigma_{stop},\tau_{stop}$ as their point estimates $E\left(\mu_{stop}|x\right)\to \mu_{stop},E\left(\sigma_{stop}|x\right)\to \sigma_{stop},E\left(\tau_{stop}|x\right)\to \tau_{stop}$ in the second stage of meta-analysis. We conduct this stage with 3 chains with 5000 burn in out of 100,000 simulations in WINBUGS1.4 software [43]. Finally, we consider the mean of posterior estimates $\mu_{\mu_{stop}},\mu_{\sigma_{stop}},\mu_{\tau_{stop}}$ in the second stage as estimates of $\vec{\theta_{S}}=\bar{\theta_{S}}=\bar{\theta_{T}}=\left(\bar{\mu_{T}},\bar{\sigma_{T}},\bar{\tau_{T}}\right)$, respectively, for the case of overall data S. We repeat this process for the case of type A SST data and type B SST data for estimation of $\bar{\theta_{A}}=\left(\bar{\mu_{A}},\bar{\sigma_{A}},\bar{\tau_{A}}\right),\bar{\theta_{B}}=\left(\bar{\mu_{B}},\bar{\sigma_{B}},\bar{\tau_{B}}\right)$, respectively.
Stage (1)


DataIndividual Priors

$GORT∼ExG(\mu_{go},\sigma_{go},\tau_{go})$
$SRRT∼ExG\left(\mu_{go},\sigma_{go},\tau_{go},\mu_{stop},\sigma_{stop},\tau_{stop},SSD\right)I_{\left[1,1000\right]}^{+}$   
$\mu_{go},\sigma_{go},\tau_{go}∼U\left[10,2000\right]$$SSRT∼ExG\left(\mu_{go},\sigma_{go},\tau_{go},\mu_{stop},\sigma_{stop},\tau_{stop},SSD\right)I_{\left[1,1000\right]}^{+}$$\mu_{stop},\sigma_{stop},\tau_{stop}∼U\left[10,2000\right]$

Stage (2)


DataPriors

${\left(\mu_{stop},\sigma_{stop},\tau_{stop}\right)}^{\prime }∼MVN\left(M_{3\times 1},\sum_{3\times 3}\right)$  

$\rho_{\mu ,\sigma },\rho_{\mu ,\tau },\rho_{\sigma ,\tau }∼U\left[-0.99,+0.99\right]$

$\mu_{stop}∼N\left(\mu_{\mu_{stop}},\sigma_{\mu_{stop}}^{2}\right)$

$\mu_{\mu_{stop}},\beta_{20},\beta_{30}∼N\left(0,1000\right)$

$\sigma_{stop}|\mu_{stop}∼N\left(\mu_{\sigma_{stop}},\sigma_{\sigma_{stop}}^{2}\right)$

$\sigma_{\mu_{stop}},\sigma_{\sigma_{stop}},\sigma_{\tau_{stop}}∼N\left(0,10\right)I_{\left[0,1000\right]}^{+}$

$\tau_{stop}|\left(\mu_{stop},\sigma_{stop}\right)∼N\left(\mu_{\tau_{stop}},\sigma_{\tau_{stop}}^{2}\right)$


$\mu_{\sigma_{stop}i}=\beta_{20}+\beta_{21}.\mu_{stop}i$


$\mu_{\tau_{stop}i}=\beta_{30}+\beta_{31}.\mu_{stop}i+\beta_{32}.\sigma_{stop}i$



**Step** (2): Paired Samples Parametric Distribution Test (PSPDT) 

This proposed test can be considered as a distributional counterpart of the paired z-test in the real numbers. Using overall estimates in Step (1), we then conduct the following paired samples parametric distribution test hypothesis testing for K=44 at the 5% significance level:

(17) $$\begin{array}{ccc} H_{0} & : & SSRT_{O.Single}\left(\vec{\theta_{S}}\right){=}_{st}SSRT_{O.Mixture}\left(\vec{\theta_{M}}\right),\\ H_{1} & : & SSRT_{O.Single}\left(\vec{\theta_{S}}\right){<}_{st}SSRT_{O.Mixture}\left(\vec{\theta_{M}}\right). \end{array}$$

where the Two-Sample Kolmogorov–Smirnov Statistics $D_{\left(n,m_{k}\right)}$ for the kth (1≤k≤K) comparisons of the simulated distributions in (17), the following average two-samples KS statistics were considered as the test statistics for the comparison of distributions in the test of (17):

(18) $$\bar{D_{n,m}}=\frac{1}{K}\sum_{k=1}^{K}D_{n,m_{k}}$$

We reject the null hypothesis $H_{0}$ in favour of alternative hypothesis $H_{1}$ at given α-level (e.g., 0.05) whenever

(19) $$\bar{D_{n,m}}>c\left(\alpha \right)\sqrt{\frac{1}{m}+\frac{1}{n}}:c\left(\alpha \right)=\sqrt{\frac{-1}{2}ln\left(\frac{1}{2}\right)},\alpha =0.05,n=m=96.$$

The Two-Sample K–S test analysis was conducted with R3.4.3 software as before. The hypothesis testing in (17) were repeated for other comparisons between cluster type SSRT indices including $SSRT_{A}$ vs. $SSRT_{Single}$, $SSRT_{B}$ vs. $SSRT_{Single}$ and $SSRT_{B}$ vs. $SSRT_{A}$.

**Remark** **3.** *The test (17) with ${<}_{st}$ replaced by $\neq_{st}$ for the degenerate case of K=1 reduces to the usual two samples K-S test at the individual level (15).*

## 3. Results

The results are divided into three subsections. In Section 3.1, we calculated the posterior mean ex-Gaussian parameter estimations of cluster type-A, cluster type-B, single and mixture SSRT distribution. Then, using them we compared the descriptive and shape statistics, including skewness and kurtosis across cluster type indices. Next, in Section 3.2, we compared single SSRT and mixture SSRT distributions in stochastic order at two levels: (i) the individual level and (ii) the population level. For the individual level, we applied IBPA, and for the population level, we used TSBPA. Finally, in Section 3.3, we compare the comparison results for the descriptive statistics and the entire SSRT distribution in terms of the cluster weights ($W_{A}$).

### 3.1. Descriptive and Shape Statistics

This section includes two sets of descriptive results: First, the results for cluster-type related mean and standard deviation of the Ex-Gaussian SSRT. Second, the results for cluster-type-related shape statistics skewness and kurtosis of the involved random variables. Throughout these results, as it is shown in Figure 4, the parameters (μ,σ,τ) refer to the mean posterior estimates of the random variables (μ,σ,τ) in the first stage of TSBPA, respectively (see Appendix B). The descriptive and shape statistics were calculated using these quantities and Equations (9) and (10).

Table 2 presents the descriptive results for the type-A, type-B, single and mixture fitted SSRT Ex-Gaussian random variable using IBPA (See Appendix B for three pramaeter estimates across three clusters). As it is shown, there is no significant difference between mean and standard deviation between cluster type SSRTs(type-B vs. type-A). However, the mentioned list of both cluster types of SSRTs is significantly larger than the single SSRT. Therefore, we conclude at this stage that the mean of mixture SSRT is significantly larger than the one of single SSRT. This result is consistent with the frequentist approach [15]. However, it is observed that the variance has significantly increased, and consequently, the precision has significantly decreased. We remind the reader that there are two evidences for violation of the assumption of equal impact of the preceding trial type (go/stop) on the current stop trial SSRT: First, despite the non-significant results presented in Table 2 (Panel (b): Type B vs. Type A) the mean type-B SSRT has a non-identity linear relationship with mean type-A SSRT ($mean.SSRT_{Bi}=\beta_{0}+\beta_{1}.mean.SSRT_{Ai}+\epsilon_{i}:\epsilon_{i}∼N\left(0,\sigma_{e}^{2}\right),\beta_{0}=96.2,$ $\left(95\%CI=\left(4.0,188.4\right)\right);\beta_{1}=0.53\;\left(95\%CI=\left(0.06,1.0\right)\right)$). Otherwise, such a relationship must be identity linear (i.e., $\beta_{0}=0,\beta_{1}=1$). Second, the mean and standard deviation of type-A SSRT and type-B SSRT are significantly different from those of single SSRT. Otherwise, all these descriptive statistics would have been equal across type-A, type-B and type-S single SST clusters.

**Figure 5:**
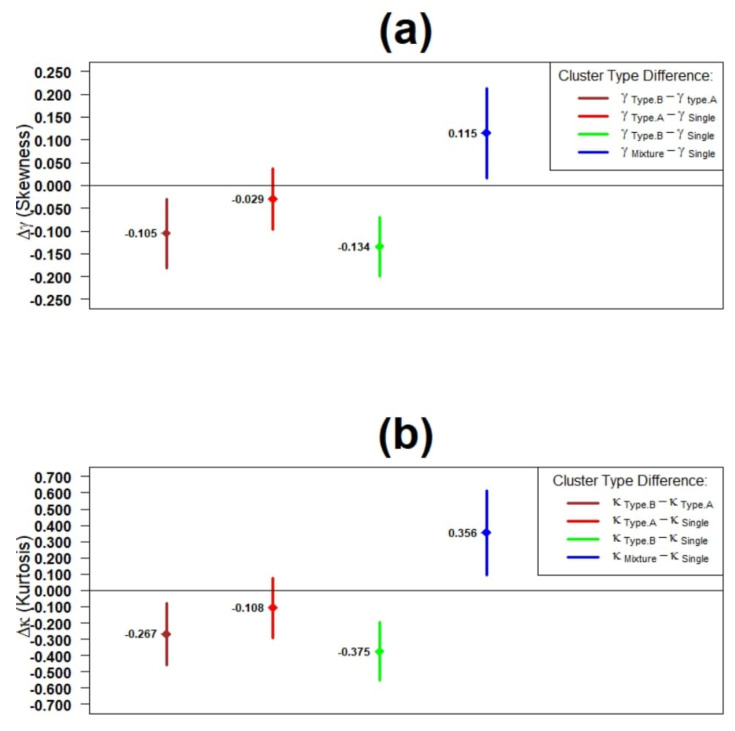
Plot of statistics difference of fitted IBPA ex-Gaussian SSRT random variable by cluster type (*n* = 44): (**a**) skewness and (**b**) kurtosis.

Figure 5 shows the difference between skewness and kurtosis of fitted IBPA Ex-Gaussian SSRT random variables by cluster type. As shown in Figure 5a, while each of Mixture SSRT components has smaller or equal skewness versus the Single SSRT, upon combination into Mixture SSRT, the resultant Mixture SSRT has significantly larger skewness compared to the Single SSRT. Similar results hold for the case of kurtosis as shown in Figure 5b.

Given summary statistics and shape statistics comparison results between single SSRT and mixture SSRT, one naturally considers comparing their associated distributions. In the next section, we deal with this topic.

### 3.2. Bayesian Mixture SSRT Estimation and Comparisons

This section deals with individual and overall level estimations of Single SSRT and Mixture SSRT and their usual stochastic order comparisons.

Table 3 presents the results of the two-sample KS hypothesis test at the individual level given by (15) by direction and *p*-values for the sample of 44 subjects based on IBPA. Similar hypothesis testing is conducted replacing ≠ with < and > in an alternative test. With two participants exception (case 34 case 37), the result shows that controlling for Family-Wise Error Rate(FWER) with Bonferroni’s correction (*p*-value = 0.05/3 = 0.0166) the single SSRT is smaller than the mixture SSRT in stochastic order. This result is consistent with the direction of constant index SSRT results [15].

We test the hypothesis (17) from TSBPA given uninformative priors for an overall conclusion using a paired samples parametric distribution test. The choice of TSBPA rather than HBPA was out of consideration for pairwise non-zero correlations in the second stage of the analysis. One key missing characteristic in the HBPA is the relaxing assumption of zero correlation of mean posterior parameters at the individual level. This assumption is violated given cluster-S SSRT mean posterior parameters Pearson correlations $\rho_{\mu \sigma }=0.20,\rho_{\mu \tau }=0.64,\rho_{\sigma \tau }=0.66;$ cluster-A SSRT mean posterior parameters Pearson correlations $\rho_{\mu \sigma }=0.52,\rho_{\mu \tau }=0.81,\rho_{\sigma \tau }=0.74;$ and, cluster-B SSRT mean posterior parameters Pearson correlations $\rho_{\mu \sigma }=0.69,\rho_{\mu \tau }=0.95,\rho_{\sigma \tau }=0.80$. Table 4 presents the results of the paired samples parametric distribution test using TSBPA:

As we observe from Table 4, the results are conclusive. The single SSRT is (provisionally) smaller than cluster type-A SSRT, cluster type-B SSRT (*p*-value < 0.0562) and Mixture SSRT. Furthermore, given consideration of mean of SSRT distribution as its point index estimation, the result regarding the comparison of single SSRT versus Mixture SSRT is consistent with the direction of the frequentist results as

$$SSRT_{Single}^{c}=E\left(SSRT_{Single}\right)=151.5<195.2=E\left(SSRT_{Mixture}\right)=SSRT_{Weighted}^{c}.$$

**Figure 6:**
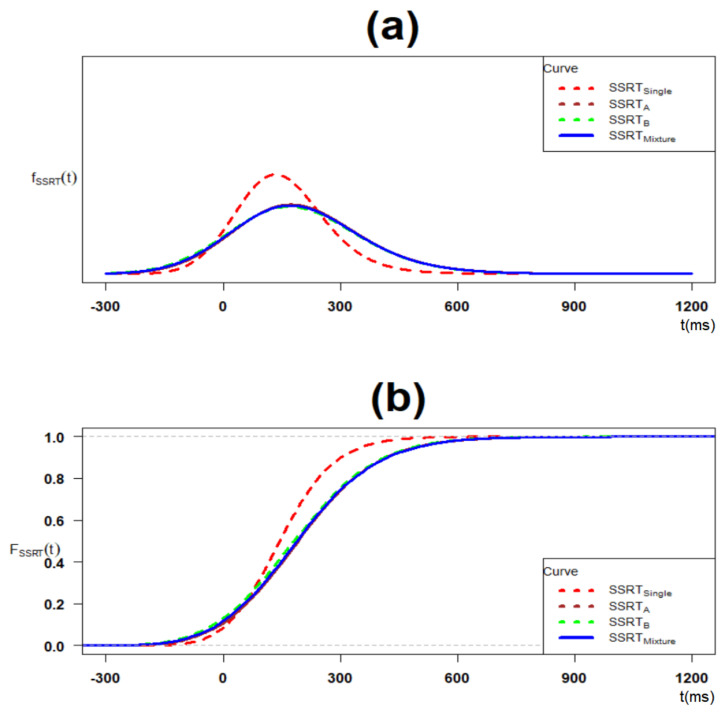
The density and cumulative distribution function (CDF) of overall sample cluster type SSRT, single SSRT and mixture SSRT with Ex-Gaussian parametric distribution: (**a**) density and (**b**) cumulative distribution function.

Figure 6 shows the plot of the overall density and cumulative distribution function of cluster type SSRTs with overall TSBPA parameter estimates given in Table 4. As it is observed in Figure 6b, while there is no such distinction between cumulative distributions of cluster type-A SSRT, cluster type-B SSRT, and Single SSRT, the cumulative distribution of single SSRT is clearly on the left side of that of Mixture SSRT.

In this and the previous section, we considered the cluster type weight $\left(W_{A}\right)$ in its fixed individual values. In the next section, we study its role in the comparison results as a critical variable on its own.

### 3.3. The Role of Cluster Type Weights in the Comparisons

This section compares the descriptive statistics of mean, variance and the entire distribution of SSRT indices in terms of individual optimal weights. By definition, the optimal weight $W_{A}$ is the most natural weight given the independence of assignment of stop or go process to the given trial [14]. The following proposition determines the values of the optimal weight [14]:

**Proposition** **1.** *The weight $W_{A}=0.75\left(W_{B}=0.25\right)$ is the optimal weight given independence of assignment of stop or go process to the given trial in the tracking SST data with proportion of 25% of stop trials.*

Note that the fitted ExG parameters θ=(μ,σ,τ) in each cluster type SST data are independent of the weights $W_{A}.$ This result is because the fitted ExG parameters for the SSRT are independent of the stop trials’ proportion. Thus, from the weight $W_{A}$ (as the result of the equality in Proposition 1). Given this result, we discuss the impact of cluster type weights on average disparities of mean SSRT estimates and the variance SSRT estimates as follows.

First, to study the impact of individual weights on the disparities of the mean estimations across indices, we consider average differences of the new index $SSRT_{Mixture}$ mean versus the established index $SSRT_{Single}$ mean in terms of the individual weights $\left(W_{A}\right)$. The averages of the ExG parameters are taken over entire n=44 participants. Considering $W_{A}$ as the main variable, it follows that

(20) $$\begin{array}{ccc} \bar{\Delta E(SSRT)}\left(W_{A}\right) & = & \bar{E(SSRT_{Mixture})}-\bar{E(SSRT_{Single})}\\ & = & \bar{E(W_{A}\cdot SSRT_{A}+\left(1-W_{A}\right)\cdot SSRT_{B})}-\bar{E(SSRT_{Single})}\\ & = & W_{A}\cdot \bar{E(SSRT_{A})}+\left(1-W_{A}\right)\cdot \bar{E(SSRT_{B})}-\bar{E(SSRT_{Single})}\\ & = & \left(\bar{E(SSRT_{A})}-\bar{E(SSRT_{B})}\right)\cdot W_{A}+(\bar{E(SSRT_{B})}-\bar{E(SSRT_{Single})}\\ & = & \left(\left(\bar{\mu_{A}}+\bar{\tau_{A}}\right)-\left(\bar{\mu_{B}}+\bar{\tau_{B}}\right)\right)\cdot W_{A}+\left(\left(\bar{\mu_{B}}+\bar{\tau_{B}}\right)-\left(\bar{\mu_{S}}+\bar{\tau_{S}}\right)\right)\\ & = & 11.4\;\;W_{A}+56.8,\;\;\;\left(0\leq W_{A}\leq 1\right). \end{array}$$

**Figure 7:**
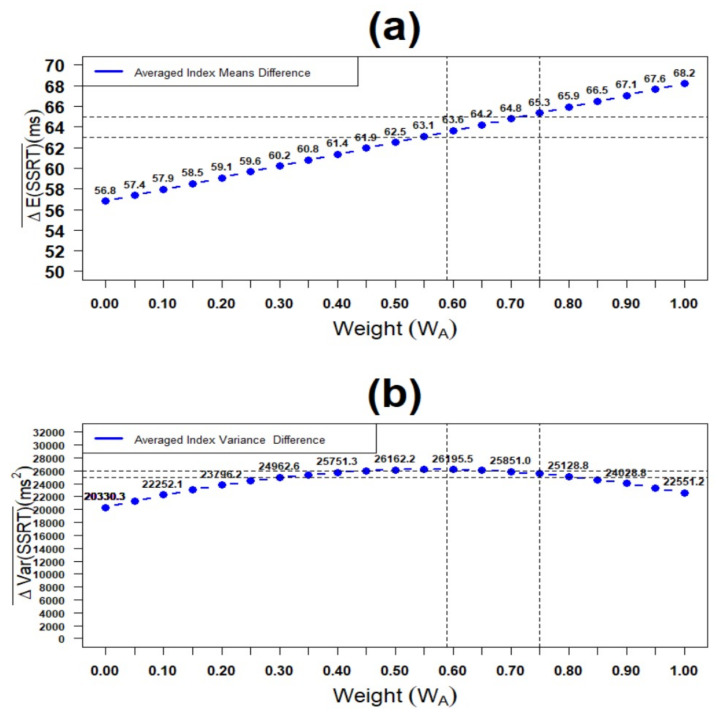
Plot of difference of SSRT index statistics ($SSRT_{Mixture}$ vs. $SSRT_{Single}$) by weight $W_{A}\left(n=44\right)$: (**a**) means and (**b**) variances.

Figure 7a presents the average disparities of mean mixture SSRT and mean single SSRT versus individual weights $W_{A}$ for the extrapolated range of [0,1]. As shown, the average difference between two index means is linear in terms of the individual weight $W_{A}$ with the minimum value of 56.8 ms (for minimum sample weight of 0.00) and maximum value of 68.2 ms (for maximum sample weight of 1.00). Furthermore, their corresponding averaged disparities equals 63.5 ms (at the overall sample weight of 0.59). Finally, the two index means’ disparities are maximised to 65.3 ms using the optimal weight of 0.75.

Second, to examine the impact of individual weights on the disparities of the variance estimations across indices, similar to the case in Section 3.2, we consider average differences of the new index $SSRT_{Mixture}$ variance versus the established index $SSRT_{Single}$ variance in terms of the individual weights $(W_{A}).$ The averages of the quadratic ExG parameters are taken over entire n=44 participants. Considering $W_{A}$ as the primary variable, it follows that

(21) $$\begin{array}{ccc} \bar{\Delta Var(SSRT)}\left(W_{A}\right) & = & \bar{Var(SSRT_{Mixture})}-\bar{Var(SSRT_{Single})}\\ & = & \bar{Var(W_{A}\cdot SSRT_{A}+\left(1-W_{A}\right)\cdot SSRT_{B})}-\bar{Var(SSRT_{Single})}\\ & = & -\bar{{\left(E\left(SSRT_{A}\right)-E\left(SSRT_{B}\right)\right)}^{2}}\cdot W_{A}^{2}\\ & & +(\bar{{\left(E\left(SSRT_{A}\right)-E\left(SSRT_{B}\right)\right)}^{2}}\\ & & +\bar{\left(Var\left(SSRT_{A}\right)-Var\left(SSRT_{B}\right)\right)})\cdot W_{A}\\ & & +\bar{Var\left(SSRT_{B}\right)-Var\left(SSRT_{Single}\right)}\\ & = & -\bar{{\left(\mu_{A}+\tau_{A}-\mu_{B}-\tau_{B}\right)}^{2}}\cdot W_{A}^{2}\\ & & +\left(\bar{{\left(\mu_{A}+\tau_{A}-\mu_{B}-\tau_{B}\right)}^{2}}+\bar{\left(\sigma_{A}^{2}+\tau_{A}^{2}-\sigma_{B}^{2}-\tau_{B}^{2}\right)}\right)\cdot W_{A}\\ & & \bar{\sigma_{B}^{2}+\tau_{B}^{2}-\sigma_{S}^{2}-\tau_{S}^{2}}\\ & = & -18885.9\;\;W_{A}^{2}+21106.8\;\;W_{A}+20330.3,\;\left(0\leq W_{A}\leq 1\right). \end{array}$$

In particular, the average variance differences attains its maximum value at

$$W_{A}=\frac{\bar{{\left(\mu_{A}+\tau_{A}-\mu_{B}-\tau_{B}\right)}^{2}}+\bar{\left(\sigma_{A}^{2}+\tau_{A}^{2}-\sigma_{B}^{2}-\tau_{B}^{2}\right)}}{2\bar{{\left(\mu_{A}+\tau_{A}-\mu_{B}-\tau_{B}\right)}^{2}}}\approx 0.56.$$

Figure 7b presents the average disparities of mixture SSRT variance and single SSRT variance versus individual weights $W_{A}$ for the extrapolated range of [0,1]. As shown, the average difference between two indices variances follows a quadratic increasing–decreasing pattern in terms of the weights $W_{A}$ with maximum values attained closed to $W_{A}\approx 0.56.$ Next, the disparities for the optimal weight $W_{A}=0.75$ is smaller than that of population weight $W_{A}=0.59.$ However, across all weights spectrum, the average SSRT variance differences are positive, showing that the new mixture SSRT index has higher variance than the current single SSRT index. Consequently, its precision is smaller.

**Figure 8:**
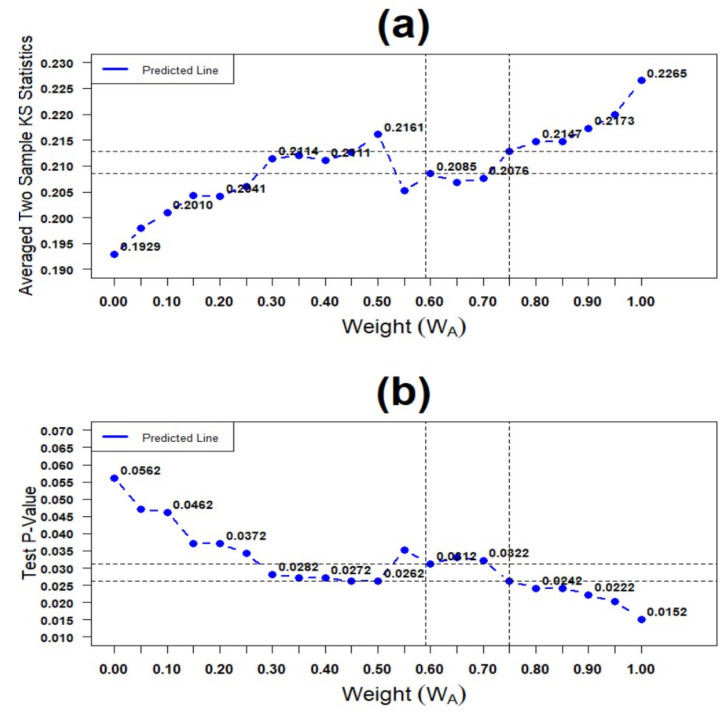
Plot of overall test results of single SSRT versus mixture SSRT: (**a**) Two-sample KS test statistics and (**b**) cut-off point of the test *p*-value.

Finally, to explore the impact of cluster type weights $\left(W_{A}\right)$ on the overall SSRT distributions comparison results for the hypothesis testing (17), we considered the averaged two-sample KS test statistics S as a function S of weights $S=S(W_{A})$ and calculated the corresponding *p*-values. Figure 8 presents the results in terms of the weights. As shown in Figure 8b, for almost all ranges of the weights $W_{A}$, the single SSRT is significantly smaller than the mixture SSRT in stochastic order. Next, the disparity is the weakest when $W_{A}$ = 0 with the corresponding *p*-value = 0.0562, and it is the strongest when $W_{A}$ = 1 with the corresponding *p*-value = 0.0152. Finally, the disparity at the optimal weight $W_{A}$ = 0.75 is more potent than that of population weight $W_{A}$ = 0.59 with corresponding *p*-values of 0.0262 and 0.0312, respectively.

## 4. Discussion

### 4.1. Present Work

This study presented a mixture Bayesian parametric approach for a more illuminating SSRT distribution estimation by considering two subtype SST cluster information suggesting a new estimation of the SSRT distribution. Furthermore, it introduced two novel statistical methodologies accompanied by their empirical applications: TSBPA and PSPDT. It was hypothesised that considering cluster type information in the new mixture SSRT distribution calculations would impact the estimation of SSRT distribution. This yields to a distributional counterpart to the case of constant index SSRT [14,15].

The results confirmed the hypothesis through three observations:

1. The descriptive and shape statistics
2. The distributional comparisons at the individual level and the population level
3. With the validity of the results in the first two observations across the entire spectrum of the weights

Similar to the constant index SSRT [15], in most cases, the mixture SSRT is different from the single SSRT in shape statistics and the stochastic order. However, in two special distinct cases, they are the same: (i) type A cluster SST is empty $\left(W_{A}=0\right)$ and (ii) type B cluster SST is empty $(W_{A}=1).$

This study confirmed that SSRT depends on non-horse race-related factors in each round of SST experimental trial, such as memory aftereffects and proportion of cluster type stop trials. It has shed light on the preparation aspect of choice stop signal reaction times by treating the previous trial type’s aftereffects as memory in the two-state mixture model [31]. Besides, given that skewness of the RT distributions increases with memory involvement (versus a perceptual decision) [44], the increase in reported skewness in the mixture SSRT versus single SSRT confirms that the proposed mixture model successfully captures the memory involvement in the decision process [44]. Furthermore, as in the context of the horse race independent model, an increase in kurtosis of SSRT is proportional to more extreme values of the right tail of SSRT distribution. Therefore, this causes a higher probability of failed inhibition in the stop trials(and vice versa). Next, the increase in reported kurtosis in the mixture SSRT versus single SSRT gives evidence that the proposed mixture model optimally uses the information given by pre-pushed failed inhibitions in the stop trials in the estimation of SSRT distribution.

This study’s findings for the SSRT distribution were consistent with the constant index SSRT when considering the impact of sub-cluster types in the estimations [14,15]. In detail, there were consistent results between the usual comparison of the single SSRT and weighted SSRT (as constant indices) and the stochastic comparison of the single SSRT and mixture SSRT (as non-constant random variables). Indeed, we found that

(22) $$\begin{array}{ccc} SSRT_{Single}^{c} & < & SSRT_{Weighted}^{c}, \end{array}$$

(23) $$\begin{array}{ccc} SSRT_{Single} & {<}_{st} & SSRT_{Mixture}. \end{array}$$

On the one hand, if we look at the two sides of the Equation (22) as degenerate random variables, we are led to Equation (23). On the other hand, if we take expectations from both sides of Equation (23), we are led to Equation (22).

This study’s novel statistical methodological contributions involved the Two-Stage Bayesian Parametric Approach (TSBPA) and the Paired Samples Parametric Distribution Test (PSPDT). TSBPA’s advantage was that it considers the underlying non-zero correlation between estimated mean posterior parameters at the first stage in the second stage’s final calculations. This feature is neglected in HBPA. PSPDT offered a novel method to compare paired sets of parametric random variables using the two-samples KS test. An application of both proposed methods was provided in this study.

There are limitations in the current study. First, the sample size was relatively small (n=44). To show more precise comparisons, larger sample size is needed. Second, the TSBPA assumes a multivariate normal distribution form for the mean posterior parameters at the second stage, which may not hold. Third, in TSBPA, when comparing overall Mixture SSRT and overall Single SSRT, there is no specific restriction on the simulation sample sizes in Equation (18). Here, while we set the sample sizes to n=m=96 (the SST data trials size), there could be other choices. Fourth, the two-sample Kolmogorov–Smirnov test has low sensitivity in the tail of the distributions when comparing them. One may consider other metrics for comparison purposes [45]. Finally, given the structure of the equations for the shape statistics (skewness and kurtosis) in terms of the cluster weights $(W_{A}),$ unlike the descriptive statistics in Section 3.3, there was no simple closed form for the averaged differences of the new index $SSRT_{Mixture}$ skewness (or kurtosis) versus the established index $SSRT_{Single}$ skewness (or kurtosis) in terms of the individual weights $(W_{A}).$ Similar to the descriptive statistics, the existence of such a simple closed formula would shed more light on the average disparities of skewness and kurtosis of the two indices across a spectrum of the individual weights.

### 4.2. Future Work

The proposed approach in modelling the SSRT distribution in this study should be replicated in future research in several different directions. This further work may shed light on further unknown corners. New work includes (i) considering the larger number of SST trials, (ii) examining the order of trials, (iii) expanding these methods to other clinical populations, (iv) considering trigger failures in the modelling, (v) interpreting the shape statistics and (vi) estimating signal respond reaction times (SRRT).

First, research has recommended that reliable estimates of SSRT for adults requires 200 SST trials with 50 stops [16]. Therefore, the current work’s approach needs to be replicated for SST data with 400 trials, including sub-cluster types of 200 trials with 50 stops for confirmation and generalisation purposes.

Second, additional research to this study must address the presumption of equal impact in the order of trials for the same cluster type weights $W_{A}.$ For example, for the case of $W_{A}=1,$ one may consider two schemes within the study of 96 SST trials: In the first scheme, trials numbered 2k(1≤k≤25) are stop trials. In the second scheme, trials numbered 98−2k(1≤k≤25) are the stop trials. There is no known study investigating if, in the same participant, these schemes lead to the same $SSRT_{Mixture}$ or not.

Third, after this study, the work should apply the proposed $SSRT_{Mixture}$ to study the inhibitory deficiency in different clinical groups such as ADHD, OCD, autism and schizophrenia. The application is in terms of descriptive statistics, shape statistics and the differential disparities across these clinical groups.

Fourth, there are trigger failures that impact the estimations [27]. Given the probability of trigger failures (TF) of $P_{T}\left(TF\right)$, $P_{A}\left(TF\right)$ and $P_{B}\left(TF\right)$ for the overall SST data, cluster-A SST data and cluster-B SST data, respectively, there remains an open question on their relationships and the impact of the cluster type trigger failures in the estimations of the $SSRT_{Mixture}$ and on the above results. The results of such consideration generalise this study’s findings in terms of trigger failures and control them in order to eliminate a potential confounding variable, trigger failure status.

Fifth, this study merely reported and compared the shape statistics for skewness and kurtosis across the cluster type SSRTs, single SSRT and the mixture SSRT distributions. There is a need to investigate these shape statistics’ psychiatric and psychopathological interpretations given the ex-Gaussian parametric distribution assumption.

Finally, this study and the earlier study in [15] addressed the estimation of stop signal reaction times (SSRT) in the case of the violated assumption of similar aftereffects of the prior trial type. It is plausible to conduct a counterpart investigation on to the estimation of the signal respond reaction times (SRRT) constant index and distribution.

### 4.3. Conclusions

There has been a great deal of interest in the aftereffects of inhibition on the estimation of SSRT in the SST literature from the early 1990s. This study addressed the problem in part and presented a two-state mixture model of SSRT distribution by considering the prior trial type with results consistent with the constant SSRT index results in the literature [15]. The results were consistent across constant index and non-constant random variable contexts in terms of the algebraic directions of the comparisons. Moreover, more information was used from the same SST data in the newly proposed mixture estimation method versus the current single estimation method. The vital assumption introduced in this work was relaxed in the newly proposed mixture estimation method. Given these advantages, we recommended considering mixture SSRT distribution $\left(SSRT_{Mixture}\right)$ as the most informative estimation of the latency of stopping.

## Figures and Tables

**Table 1:** Partition of stop task (SST) data to Type A SST data and Type B SST data given previous trial type (go/stop) [15].

Data		Previous Trial	
		**Go**	**Stop**
Current Trial	Go	$Go_{A}$	$Go_{B}$
	Stop	$Stop_{A}$	$Stop_{B}$

**Table 2:** Descriptive results for mean and standard deviation of fitted IBPA ex-Gaussian distribution to SSRT given cluster type (*n* = 44).

**(a) Descriptive Results**		
	**Statistics (Mean (95%CI))**	
**Cluster Type**	**Mean**	**St.d**
Type S	196.8	157.8
	(173.5, 220.1)	(139.4, 176.2)
Type A	265.0	217.7
	(235.8, 294.2)	(199.1, 236.2)
Type B	253.6	213.2
	(222.9, 284.2)	(195.7, 231.0)
**(b) Two Sample** ***t*** **test**		
	**Statistics (Mean (95% CI))**	
**Comparison**	**Mean**	**St.d**
Type B vs. Type A	−11.4	−4.4
	(−53.5, 30.7)	(−33.5, 24.7)
Type B vs. Type S	56.8 ***	55.5 ***
	(32.2, 81.4)	(35.4, 75.7)
Type A vs. Type S	68.2 ***	59.9 ***
	(48.3, 88.1)	(44.4, 75.4)
Type M vs. Type S	63.7 ***	71.4 ***
	(57.2, 70.1)	(62.5, 80.3)

Notes: $\bar{W_{A}}=0.59,$ *** *p*-value <0.0005.

**Table 3:** Two-sample Kolmogorov–Smirnov test results for the Single SSRT distribution versus Mixture posterior SSRT distribution (*n* = 44).

		Alternative		Hypothesis		
	**Unequal**		**Greater**		**Less**	
#	**Statistics**	***p*** **-Value**	**Statistics**	***p*** **-Value**	**Statistics**	***p*** **-Value**
1	0.2708	0.0017	0.0417	0.8465	0.2708	0.0009
2	0.2604	0.0029	0.0208	0.9592	0.2604	0.0015
3	0.3333	0.0001	0.0729	0.6002	0.3333	0.0001
4	0.3438	0.0001	0.0417	0.8465	0.3438	0.0001
5	0.2396	0.0079	0.0312	0.9105	0.2396	0.0040
6	0.2812	0.0009	0.0417	0.8465	0.2812	0.0005
7	0.3021	0.0003	0.0729	0.6002	0.3021	0.0002
8	0.2188	0.0200	0.0312	0.9105	0.2188	0.0101
9	0.3646	0.0001	0.0521	0.7707	0.3646	0.0001
10	0.2396	0.0079	0.0312	0.9105	0.2396	0.0040
11	0.3229	0.0001	0.0417	0.8465	0.3229	0.0001
12	0.3229	0.0001	0.0000	1.0000	0.3229	0.0001
13	0.3021	0.0003	0.0208	0.9592	0.3021	0.0002
14	0.2396	0.0079	0.0104	0.9896	0.2396	0.0040
15	0.3229	0.0001	0.0625	0.6873	0.3229	0.0001
16	0.2500	0.0048	0.0312	0.9105	0.2500	0.0025
17	0.3229	0.0001	0.0417	0.8465	0.3229	0.0001
18	0.3542	0.0001	0.0000	1.0000	0.3542	0.0001
19	0.2604	0.0029	0.0417	0.8465	0.2604	0.0015
20	0.2604	0.0029	0.0312	0.9105	0.2604	0.0015
21	0.3854	0.0001	0.1042	0.3529	0.3854	0.0001
22	0.3438	0.0001	0.0417	0.8465	0.3438	0.0001
23	0.3646	0.0001	0.1146	0.2835	0.3646	0.0001
24	0.3021	0.0003	0.0104	0.9896	0.3021	0.0002
25	0.4896	0.0001	0.0833	0.5134	0.4896	0.0001
26	0.2604	0.0029	0.0104	0.9896	0.2604	0.0015
27	0.3958	0.0001	0.0729	0.6002	0.3958	0.0001
28	0.2708	0.0017	0.0208	0.9592	0.2708	0.0009
29	0.2396	0.0079	0.0104	0.9896	0.2396	0.0040
30	0.3750	0.0001	0.0729	0.6002	0.3750	0.0001
31	0.4062	0.0001	0.0312	0.9105	0.4062	0.0001
32	0.2500	0.0048	0.0208	0.9592	0.2500	0.0025
33	0.2500	0.0048	0.0208	0.9592	0.2500	0.0025
34	0.1562	0.1923	0.0000	1.0000	0.1562	0.0960 *
35	0.2500	0.0048	0.0104	0.9896	0.2500	0.0025
36	0.3021	0.0003	0.0312	0.9105	0.3021	0.0002
37	0.1979	0.0463	0.0104	0.9896	0.1979	0.0233 *
38	0.3125	0.0002	0.0312	0.9105	0.3125	0.0001
39	0.2917	0.0005	0.0625	0.6873	0.2917	0.0003
40	0.3750	0.0001	0.0521	0.7707	0.3750	0.0001
41	0.2188	0.0200	0.0417	0.8465	0.2188	0.0101
42	0.3021	0.0003	0.0208	0.9592	0.3021	0.0002
43	0.2188	0.0200	0.0104	0.9896	0.2188	0.0101
44	0.4062	0.0001	0.0312	0.9105	0.4062	0.0001

Notes: IBPA: #Chains = 3; Simulations = 20,000; Burn-in = 5000 (for both single and mixture parameters); The sample size for K-S test for each distribution was *n* = *m* = 96; *: Exceptional case.

**Table 4:** Two-sample Kolmogorov–Smirnov test results for the cluster type SSRT distributions in the hypothesis test (17) (*n* = 44).

Comparison	Statistics	*p*-Value
$SSRT_{Single}$ vs. $SSRT_{Mixture}$	0.2095	<0.0312
$SSRT_{Single}$ vs. $SSRT_{B}$	0.1984	<0.0562
$SSRT_{Single}$ vs. $SSRT_{A}$	0.2256	<0.0152
$SSRT_{B}$ vs. $SSRT_{A}$	0.0653	>0.9999

Note: The sample sizes for K-S test for both distributions were n=m=96. $\vec{\theta_{S}}=\left(\bar{\mu_{T}},\bar{\sigma_{T}},\bar{\tau_{T}}\right)=\left(78.4,93.9,73.1\right),$ $\vec{\theta_{A}}=\left(\bar{\mu_{A}},\bar{\sigma_{A}},\bar{\tau_{A}}\right)=\left(94.0,134.5,104.8\right),$ $\vec{\theta_{B}}=\left(\bar{\mu_{B}},\bar{\sigma_{B}},\bar{\tau_{B}}\right)=\left(90.9,142.3,99.0\right)$ and $\vec{\theta_{M}}=\left(\bar{W_{A}},\vec{\theta_{A}},\vec{\theta_{B}}\right),$ with $\bar{W_{A}}=0.59.$

## Data Availability

Not applicable.

## Abbreviations

The following abbreviations are used in this manuscript:

ADHD	Attention Deficit Hyperactivity Disorder
BEESTS	Bayesian Ex-Gaussian Estimation of Stop Signal RT distributions
BPA	Bayesian Parametric Approach
ExG	Ex-Gaussian distribution
FWER	Family-Wise Error Rate
GORT	Reaction Time in a go trial
GORTA	Reaction Time in a type A go trial
GORTB	Reaction Time in a type B go trial
HBPA	Hierarchical Bayesian Parametric Approach
IBPA	Individual Bayesian Parametric Approach
MCMC	Marco Chain Monte Carlo
OCD	Obsessive Compulsive Disorder
PSPDT	Paired Samples Parametric Distribution Test
SSD	Stop Signal Delay
SRRT	Reaction Time in a failed stop trial
SRRTA	Reaction Time in a failed type A stop trial
SRRTB	Reaction Time in a failed type B stop trial
SSRT	Stop Signal Reaction Times in a stop trial
SSRTA	Stop Signal Reaction Times in a type A stop trial
SSRTB	Stop Signal Reaction Times in a type B stop trial
SST	Stop Signal Task
TF	Trigger Failure
TSBPA	Two-Stage Bayesian Parametric Approach

## Appendix A. Sample SST Data and Its Clusters

This section presents a sample SST dataset of 48 trials with 36 go trials and 12 stop trials with initial SSD = 200 ms. The GORT and SRRT have ex-Gaussian distributions (values: −999: missing value; NA: not applicable; 0: failed inhibition; 1: successful inhibition).

**Block**	**Trial**	**GORT (ms)**	**SRRT (ms)**	**SSD (ms)**	**Trial**	**Inhibition**	**Cluster**
1	1	356.4	−999	−999	Go	NA	A
1	2	426.5	−999	−999	Go	NA	A
1	3	−999	380	350	Stop	0	A
1	4	397.2	−999	−999	Go	NA	B
1	5	−999	−999	200	Stop	1	A
1	6	−999	−999	100	Stop	1	B
1	7	283.6	−999	−999	Go	NA	B
1	8	457.3	−999	−999	Go	NA	A
1	9	361.9	−999	−999	Go	NA	A
1	10	375.7	−999	−999	Go	NA	A
1	11	478.5	−999	−999	Go	NA	A
1	12	355.9	−999	−999	Go	NA	A
1	13	−999	−999	150	Stop	1	A
1	14	300.5	−999	−999	Go	NA	B
1	15	347	−999	−999	Go	NA	A
1	16	390.3	−999	−999	Go	NA	A
1	17	327.2	−999	−999	Go	NA	A
1	18	300.4	−999	−999	Go	NA	A
1	19	382.2	−999	−999	Go	NA	A
1	20	326.9	−999	−999	Go	NA	A
1	21	−999	389.8	200	Stop	0	A
1	22	−999	445.4	200	Stop	0	B
1	23	352.4	−999	−999	Go	NA	B
1	24	403.1	−999	−999	Go	NA	A
2	1	256.4	−999	−999	Go	NA	A
2	2	426.5	−999	−999	Go	NA	A
2	3	−999	360	350	Stop	0	A
2	4	353.2	−999	−999	Go	NA	B
2	5	−999	−999	200	Stop	1	A
2	6	−999	−999	100	Stop	1	B
2	7	253.6	−999	−999	Go	NA	B
2	8	427.3	−999	−999	Go	NA	A
2	9	351.9	−999	−999	Go	NA	A
2	10	355.7	−999	−999	Go	NA	A
2	11	455.5	−999	−999	Go	NA	A
2	12	335.9	−999	−999	Go	NA	A
2	13	−999	−999	150	Stop	1	A
2	14	321.5	−999	−999	Go	NA	B
2	15	322	−999	−999	Go	NA	A
2	16	340.5	−999	−999	Go	NA	A
2	17	317.2	−999	−999	Go	NA	A
2	18	303.1	−999	−999	Go	NA	A
2	19	322.9	−999	−999	Go	NA	A
2	20	316.5	−999	−999	Go	NA	A
2	21	−999	368.7	200	Stop	0	A
2	22	−999	435.2	200	Stop	0	B
2	23	342.3	−999	−999	Go	NA	B
2	24	413.5	−999	−999	Go	NA	A

## Appendix B. SSRT ExG Cluster Type Parameter Estimations

This section presents the data of mean posterior ex-Gaussian parameters estimations across trial types by IBPA given by Equation (7) and denoted by $E\left(\mu_{stop}|x\right)\to \mu_{stop},E\left(\sigma_{stop}|x\right)\to \sigma_{stop},E\left(\tau_{stop}|x\right)\to \tau_{stop}$ in Figure 4, (*n* = 44).

	μ−	**Parameter**		σ−	**Parameter**		τ−	**Parameter**	
#	$\mu_{S}$	$\mu_{A}$	$\mu_{B}$	$\sigma_{S}$	$\sigma_{A}$	$\sigma_{B}$	$\tau_{S}$	$\tau_{A}$	$\tau_{B}$
1	67.899	110.575	74.101	92.015	174.890	128.896	118.238	180.543	96.303
2	80.776	134.302	74.966	115.760	161.009	114.076	98.747	164.481	72.904
3	101.214	73.414	240.510	69.946	86.953	246.686	62.308	58.302	224.543
4	76.125	101.214	81.148	70.314	97.988	161.328	60.001	103.904	87.476
5	107.774	115.981	144.308	133.990	216.231	136.901	137.250	175.537	120.983
6	82.189	72.291	116.641	80.937	104.697	152.139	66.669	82.550	129.349
7	91.065	102.595	98.262	98.313	187.322	160.173	61.743	107.831	96.720
8	104.330	104.326	161.787	132.146	119.162	217.623	109.687	102.335	167.392
9	65.595	79.870	102.948	90.731	166.297	139.209	54.418	92.445	102.436
10	51.841	57.038	118.292	138.916	90.925	244.038	87.252	57.347	163.274
11	68.697	101.933	87.970	96.996	180.128	120.074	69.633	123.469	89.641
12	46.706	104.368	58.037	163.780	185.456	139.646	59.603	140.534	62.199
13	68.347	132.873	74.025	153.626	245.102	144.620	78.252	175.639	80.781
14	97.394	96.609	227.966	173.632	130.787	247.703	130.172	98.451	205.539
15	82.559	72.106	129.965	77.780	126.941	142.630	67.081	82.437	139.205
16	117.957	171.526	95.175	95.554	171.345	104.171	128.851	214.714	98.275
17	64.794	94.754	76.374	114.202	123.549	203.176	58.881	107.584	89.813
18	87.286	193.774	120.393	155.274	209.582	184.605	96.340	200.869	122.920
19	94.714	125.221	81.660	111.226	168.084	136.605	99.098	159.208	100.093
20	75.245	81.478	141.656	97.756	118.291	181.230	104.026	110.761	160.693
21	71.062	95.747	64.564	58.011	118.150	173.817	52.313	105.009	85.212
22	81.872	127.913	60.126	78.948	113.331	121.584	66.274	135.348	72.943
23	107.987	96.020	111.418	50.664	115.317	166.393	51.215	103.610	124.598
24	101.584	209.627	87.178	171.609	218.032	183.082	131.537	222.475	103.681
25	52.794	77.214	101.703	67.324	186.747	175.459	46.073	100.695	123.011
26	95.816	85.851	194.075	189.521	246.825	184.251	123.096	127.805	176.595
27	117.658	153.634	93.757	62.032	131.224	163.681	62.041	145.738	105.041
28	74.982	101.042	118.134	162.917	169.631	223.536	100.496	128.872	147.351
29	88.309	95.600	161.360	149.086	179.344	203.212	147.284	164.919	178.930
30	110.214	112.539	114.247	62.896	123.664	159.486	59.637	119.830	122.869
31	69.559	113.793	84.797	105.813	162.989	163.887	66.243	144.137	98.274
32	86.656	106.062	121.578	141.479	182.938	190.266	137.950	147.733	174.887
33	91.486	98.925	138.757	153.716	194.385	186.988	136.731	153.773	160.485
34	117.334	332.094	82.688	173.864	291.666	98.011	266.191	267.137	79.306
35	211.322	298.325	263.992	174.198	177.075	239.405	249.031	201.246	236.296
36	169.964	205.776	200.058	118.306	193.870	168.009	151.777	198.589	177.212
37	107.486	113.820	264.434	173.840	134.020	256.407	221.662	114.100	258.968
38	82.222	96.709	101.946	94.497	150.815	139.692	88.207	126.417	121.590
39	71.434	100.440	95.851	84.124	181.053	121.111	113.964	168.324	102.433
40	121.865	147.288	131.370	58.999	108.255	161.302	76.354	130.551	135.443
41	173.617	177.401	234.773	154.409	229.659	178.450	210.027	234.848	194.605
42	92.570	127.033	128.485	120.396	180.311	143.743	113.738	158.973	126.733
43	75.441	110.671	65.551	144.454	217.324	111.055	80.645	138.963	71.760
44	92.572	148.911	124.385	98.960	160.314	171.516	59.378	123.322	116.718
Notes: *μ_S_*, *σ_S_*, *τ_S_*: ExG SSRT parameters for single cluster SST data; *μ_A_*, *σ_A_*, *τ_A_*: ExG SSRT parameters for type-A cluster SST data; *μ_B_*, *σ_B_*, *τ_B_*: ExG SSRT parameters for type-B cluster SST data; IBPA: #Chains = 3; Simulations = 20,000; Burn-in = 5000 (for both single and mixture parameters).									
